# Not yet defect-free: the current landscape for women in computational materials research

**DOI:** 10.1038/s41524-023-01054-z

**Published:** 2023-06-03

**Authors:** Livia B. Pártay, Erin G. Teich, Rose K. Cersonsky

**Affiliations:** 1 Department of Chemistry, University of Warwick, Coventry, CV4 7AL United Kingdom; 2 Department of Physics, Wellesley College, 106 Central Street, Wellesley, 02481 MA USA; 3 Department of Chemical and Biological Engineering, University of Wisconsin - Madison, 1415 Engineering Drive, Madison, 53706 WI USA

**Keywords:** Theory and computation, Materials science

## Introduction

“I really am not seeing how women are still considered disadvantaged in our field.”

This phrase is heard all-too-often by woman scientists across many fields. And while attitudes on gender equity and parity have considerably improved in recent history, there are still measurable inequities stemming from systemic bias in the career progression, recognition, and perception of women in the sciences, including our sub-field of computational materials research.

Addressing the obstacles to equity is in the interests of all of us. Making research inclusive is critical, not only from an ethical standpoint, but also for attracting and retaining dedicated and talented minds, enhancing the vibrancy of all fields, and nurturing a diverse range of ideas. Diversity in representation and thought can increase a group’s collective intelligence, improve productivity, and improve retention in growing fields such as ours^1–3^. Representation of the entire populace is also necessary for ensuring that science is responsible and relevant to societal needs^4^.

In this perspective, we review the current challenges and barriers for women in the sciences, citing data and analyses from the literature, and including, where appropriate, contextualized anecdotes. Many of these experiences are not singular to gender, and often intersect and compound with other aspects of one’s identity, including those related to race, sexual orientation, religion, country of origin, and disability. Yet, while it may be true that improving equity along one dimension of diversity may improve the environment for everyone, it is important to both independently and intersectionally center these other dimensions in actions and initiatives, as biases against other underrepresented populations are also nuanced and deserving of attention.

Although the intersecting set of challenges faced by women in the sciences is daunting, numerous efforts are ongoing to address them at both the individual and collective levels. We close by outlining some of these successful practices to further equity in the sciences.

## How representative is the current academic landscape in computational materials research?

"During my undergraduate studies (only about a decade ago), I had one woman professor in my materials science classes and none in my computer science classes. And in these classrooms, I was one of a few women, if not the only woman, present. Because of this, my male professors often took an opportunity to single me out or ask me to relate their examples to my lived-in experience. All of this subconsciously communicated that I didn’t belong or that I had to be extraordinary to justify my presence.” (Female researcher)

We start by quantifying and contextualizing the number of women within the relevant fields of materials science and engineering (MSE), chemical engineering (ChE), physics, and chemistry, noting especially how the proportion of women drops at every rung of the career ladder (a phenomenon often referred to as the “leaky pipeline”).

**Fig. 1:**
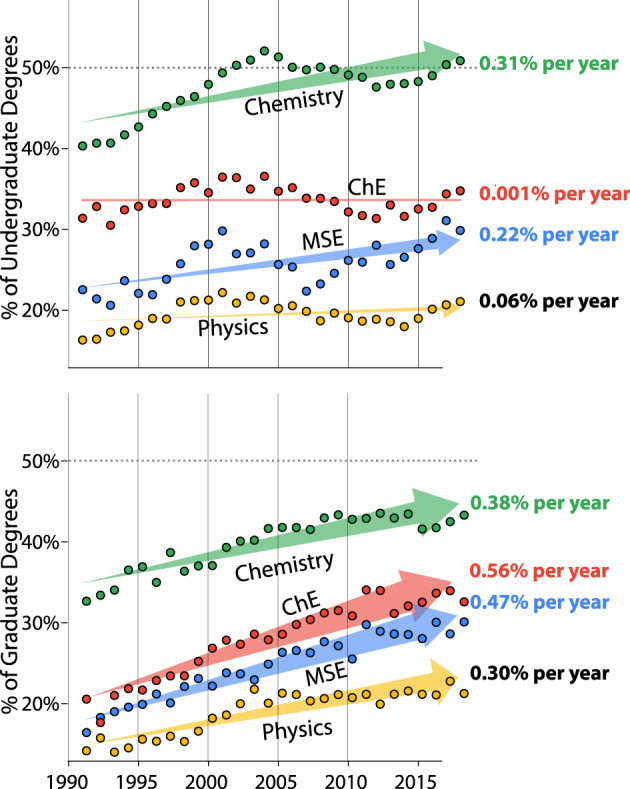
Trends in the percentage of undergraduate and graduate degrees awarded to women in the United States of America, National Center for Education Statistics^5^. Arrow widths are proportional to the rate of increase. Trendlines were determined through regularized linear regression.

Each year the US Department of Education National Center for Education Statistics (NCES) reports the number of degrees awarded by US academic institutions, separated by discipline and gender. From their reported data (covering 1992–2019)^5^, see Fig. 1, we see some improvement in undergraduate representation over the past 30 years in the chemistry and MSE fields, in which the percentage of degrees awarded to women increased by 5–10% (with chemistry recently reaching parity). Other fields, such as chemical engineering and physics, have remained somewhat stagnant in undergraduate degree representation. Across all fields, the proportion of graduate degrees awarded to women has steadily increased over the past 30 years. While we lack similarly granular data for most other countries, corresponding trends have been noted for non-US institutions^6–8^, namely that despite gains in proportional representation in related graduate degrees, the gap between male and female undergraduates in science, technology, engineering, and mathematics (STEM) disciplines often still looms large. These trends project that many **graduate programs should reach parity within the next century**; however, reaching **parity within the undergraduate populations (apart from chemistry) is likely impossible in the near future** without any meaningful changes in the current rates.

These trends reflect the disciplines at large, not solely the computationally-focused subdivisions within each field. Alarmingly, the nearby fields of **computer science and mathematics have shown a noted** **decline** **in the proportion of female graduates in the past decades**^9^, which suggests that the improvement in the representation of women in theory- and computation-focused physical sciences may be below what is suggested by the discipline-wide data.

**Fig. 2:**
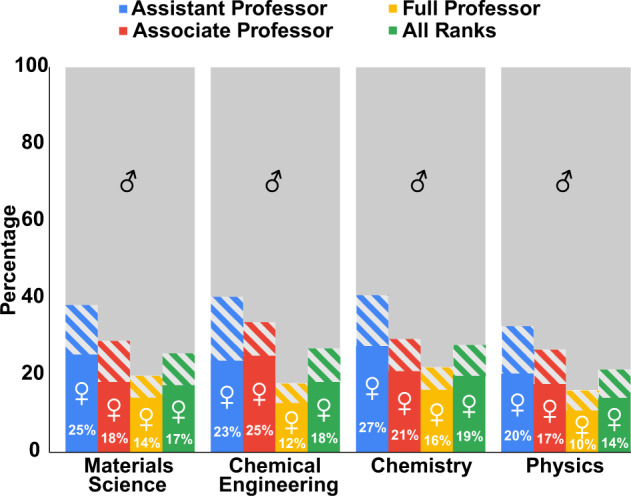
Percentages of female faculty across ranks, as reported by the Academic Analytics Research Center (https://aarcresearch.com/data). Striped bars indicate faculty of unknown gender. Here we use the American-style professorial ranks, where an “assistant” professor refers to a pre-tenure junior professor, an “associate” professor refers to a post-tenure mid-level professor, and a “full” professor refers to a senior-level tenured professor.

According to the Academic Analytics Research Center, which compiles data on academic representation and recognition, roughly 14–20% of professors (at all ranks) in these fields are women^10,11^, see Fig. 2, with the **representation dropping with professorial rank** (i.e., significantly more women are junior and mid-level professors than full professors). The proportion of women entering faculty positions in STEM has risen in recent years^12^; thus, the lack of senior women faculty could be partially explained by the retirement and turnover of historically male faculties. However, Wapman et al.^13^ showed that the **higher attrition rate of (both non-white and) female faculty members** largely contributes to the lack of diversity at higher professorial ranks and noted that strong emphasis should be placed on retaining diverse colleagues in academic departments.

## What causes and perpetuates disadvantages in research environments?

**Fig. 3:**
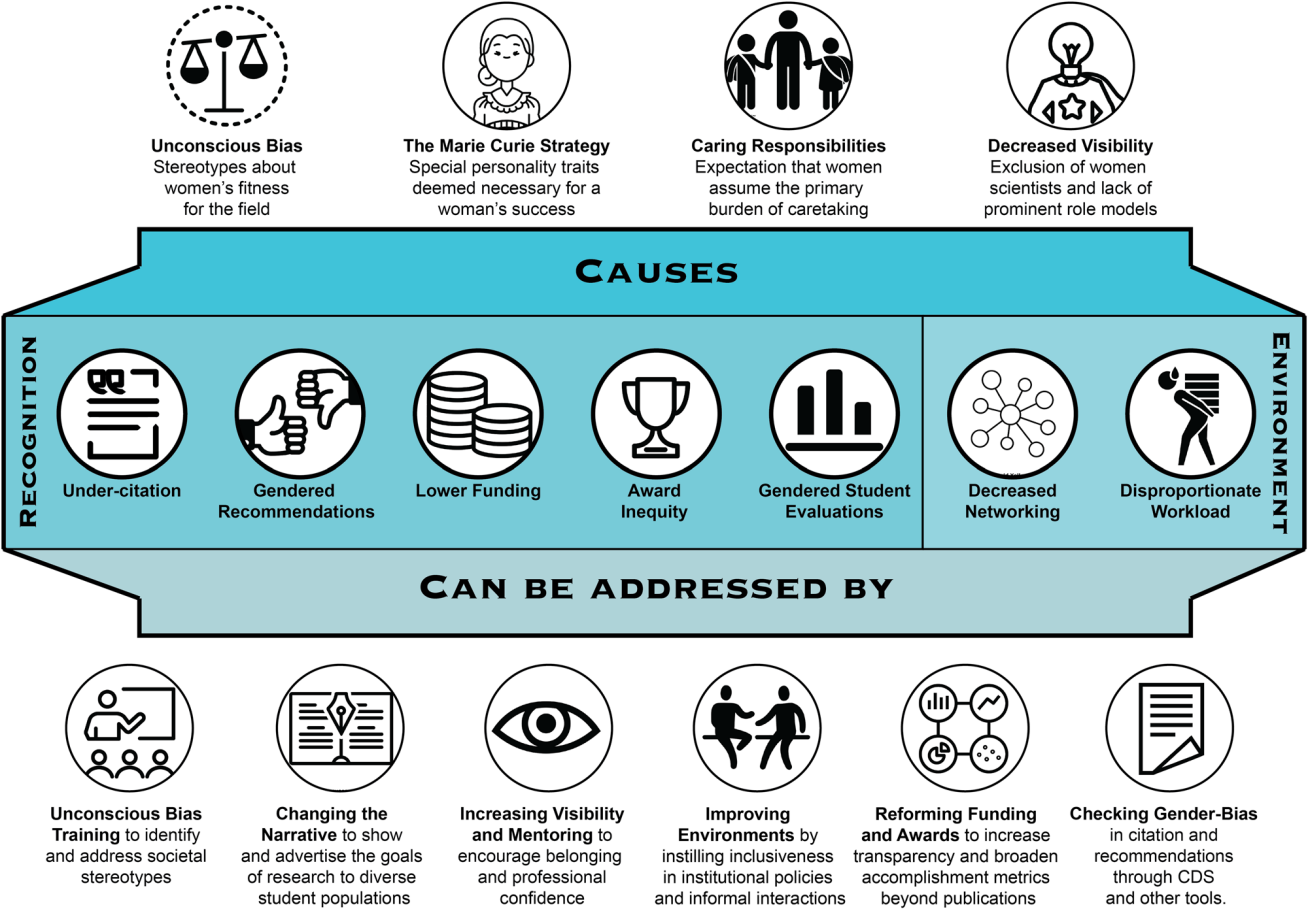
A Summary of gender inequity causes, manifestations, and responses. Many of these phenomena are interrelated, and it is important to take a multi-pronged approach to addressing the causes and manifestations of these inequities. Individual images obtained through the Noun Project (thenounproject.com).

In order to take positive actions to address the current and historical barriers faced by women in STEM environments, it is first necessary to understand the underlying causes and effects of these barriers. We hope this will not only help to raise awareness in the community, but will also serve as a concise summary and starting point for researchers committed to making a difference in transforming the research landscape. We summarize the sociological phenomena that lead to gender-based inequities, many of which can be addressed by thorough and regular unconscious bias training. These inequities can be primary and/or secondary in nature, *i.e*., disadvantages caused by both underlying stereotypes of women and by the response of society, institutions, and persons to these stereotypes. These are also summarized in Fig. 3.

### Implicit bias

“I would say the societal expectation that scientists should be non-female. This starts out as an expectation from families and school teachers and friends that young women shouldn’t become scientists so that they don’t explore the option. Then it manifests as unconscious bias or even active discrimination in evaluations of woman scientists’ work, as well as in gender-based harassment. I would say that the expectation probably stems in turn from the fact that there are not so many leading women scientists and so when people think of a scientist, they don’t think of somebody female.” (Prof. Dr. Nicola Spaldin, when asked about the biggest challenges facing women scientists)^14^

We all hold unconscious biases, shaped by the stereotypes of our wider cultural environment and experiences. While creating such stereotypes is an unavoidable consequence of human cognition, these perceptions often negatively affect women (or, more broadly, anyone adopting behavior typically associated with women^15,16^), even if there is no intention of hurt or discrimination^17^. Addressing unconscious bias is difficult and necessary for even the most allied scientist, as most of our stereotypes and schemas operate below the level of awareness.

**Fig. 4:**
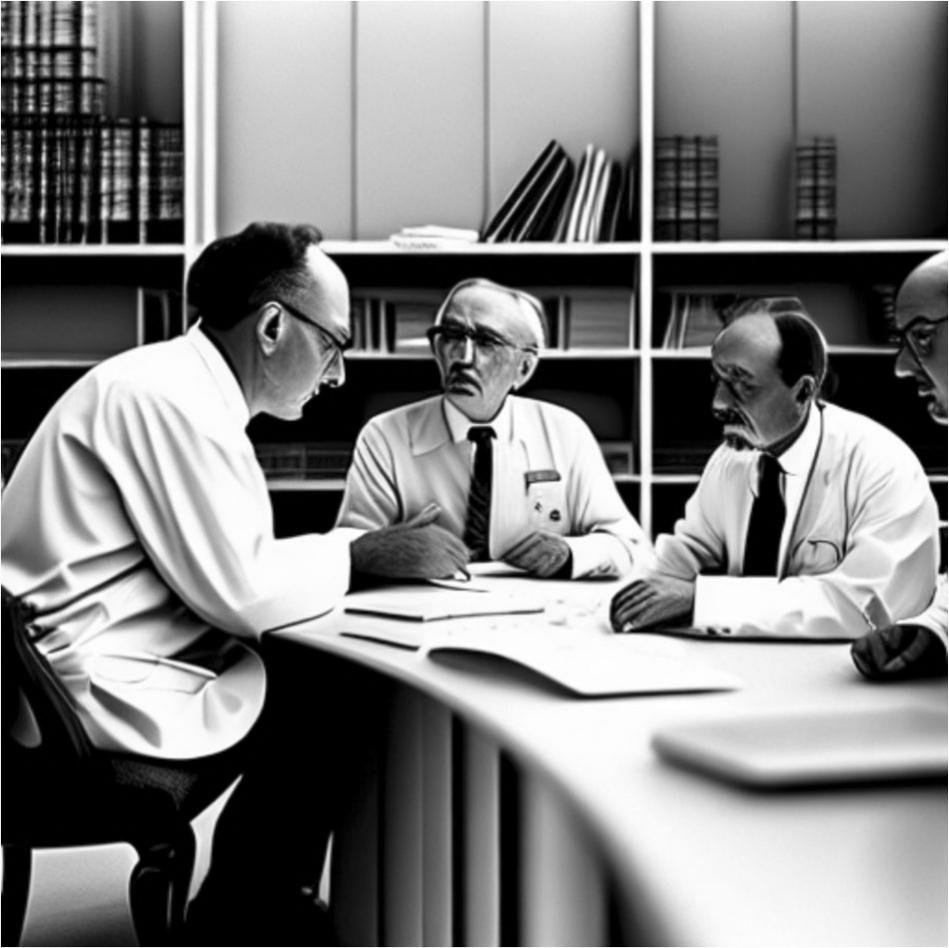
Usage of artificial intelligence. Would you use an AI tool to create graphics for your presentation or visual communications? *Four computational materials scientists having a research discussion*, as imagined by the AI tool images.ai, only depicts older white men.

Unrecognized biases mean that women often face rather different expectations than their male colleagues in the research environment^18,19^. **STEM areas (especially engineering and computing) are still widely perceived as “boys’ subjects”**^19^. These perceptions are not only ingrained in our society’s understanding of certain topics but also shape society’s **ongoing narrative about who is a scientist and what it looks like to achieve scientific progress**. For example, recent efforts have focused on training artificial intelligence (AI) on societal data in order to predict correlations^20^. If you ask a recently trending image generator^21^ for “four computational materials scientists having a research discussion”, the generator will return an image of four older white men around a table (see Fig. 4). As AI technologies become embedded in every aspect of modern life, it is pivotal that we engage in a critical discussion on how we can avoid using these tools to reinforce harmful stereotypes.

**Women’s contributions are often further underrepresented in or excluded from science textbooks**^22^, contributing to damaging stereotypes about who is (and who is not) a “scientist”^23^. The widespread perception that women lack academic excellence in comparison to their male peers exists at all levels of the science educational system^24–27^. The internalization of these stereotypes can lead to lower performance^28^ and interest^29^ among women and girls in scientific environments, thus compounding the exclusion of women from these spaces.

Women are also deemed less likely to be leaders (by both men and other women) and are less likely to be accepted as leaders^18,30^. This directly affects researchers’ success, as students are less likely to choose a female supervisor or join a group with a female primary investigator (PI), believing them to be less capable of helping one’s career progression and less prestigious to work with overall^31^.

### The Madame Curie strategy

“... the Madame Curie strategy, as it was termed earlier, of quiet but deliberate over-qualification, personal modesty, strong self-discipline, and infinite stoicism [required to assimilate into a male-dominated workplace].” (Margaret Rossiter)^32^

Despite the decades since this coinage, **the “narrow band of acceptable behavior” specially required for women’s success in science**, particularly personality traits associated with modesty and quietude, is a common theme in many women’s accounts of social obstacles to their success^33^. These expectations can both determine how women choose to comport themselves and how they are received—statistically, women are offered fewer opportunities to express their opinion than men, responses and answers given to women are often less detailed and less informative, women are more likely to be interrupted when speaking, and their ideas dismissed (then sometimes, ironically, applauded when a man raises them)^31,34^. When women are not in the extreme minority and encouraged to participate meaningfully, there is a measurable positive impact on the productivity and problem-solving capacity of a team, based on increases in conversational turn-taking and cooperativeness and improved collaborative patterns^1,35–37^.

Women also tend to underestimate their own skills, especially in environments where gendered stereotypes are active^38^, and this culminates in “biased” self-assessment and reductions in seeking out opportunities, self-promotion, and self-nomination. Successful self-promotion is a crucial tool for succeeding in a research environment and enhancing one’s visibility and perceived competence. While men usually employ this successfully, self-promoting women are found to be less likable or hireable^16,18,39–41^.

Thus **when women deviate from socially expected behaviors** and adopt traditionally male-associated communication styles, **there is a backlash**^12,42,43^, and noting these more subtle forms of discrimination are often dismissed as personal sensitivity.

### Caring responsibilities

“If you were to draw conclusions about faculty demographics from reading confidential letters, you’d conclude that only women have children (as you rarely read about family matters in letters about men).” (Judith D. Singer, quoting a reference letter she had received)^44^

Many will point to familial responsibilities as a reason for the delayed progression of women in research positions^45^. Childbearing typically occurs within the crucial and productive early stages of a research career, and women researchers are more likely than their male counterparts to limit their starting or growing of a family in order to further their career^46^. Additionally, women are twice as likely to leave their STEM research positions than men in the years following the birth of their first child, with an attrition rate of 50% compared to 25%^47^. The irony is that balancing work and family is a conundrum universal to all parents, of any gender. So **why are women with children experiencing greater career depression than men with children**^47,48^?

Despite the evolution of gender roles over the past fifty years, caring roles are still mostly assumed by women^12^, with the COVID-19 pandemic likely to have caused setbacks^49^. There are multiple arguments to be made here: (1) women choose familial obligations over scientific ambition or (2) the social norms and infrastructure of STEM institutions do not adequately support women who both want to pursue a scientific career and choose to have children or care for a relative.

The reality is a messy combination of the two—**women should always be free to choose to have children or to leave scientific careers, but some** ***have this choice made for them*** **based upon a lack of meaningful partner engagement or institutional support**. And even if a woman chooses to pursue both paths in life, the assumptions made about a woman’s commitment or responsibilities can result in further exclusion from networking and collaboration opportunities^12^, leading many women with children or caring responsibilities to feel behind their male counterparts^50^.

### Lack of role models and visibility

“I don’t think there are any role models I know who managed to balance academic career and a family and life” (Female chemistry PhD student)^11^

**Lack of female role models** negatively influences self-esteem and **prevents the formation of one’s identity as a woman scientist**^51^. The lack of visibility creates the perception that female scientists are isolated and disconnected, which discourages other women from imagining their own place in the research landscape, thereby contributing to the *leaky pipeline*^31^.

Representation at conferences can be a particularly powerful way of enhancing visibility and increasing the esteem of women scientists. At the Psi-k conference series (1000+ international participants) in 2010, only 10% of invited speakers were women; this proportion increased slightly to 16% in 2015, which still meant that at least half of the symposia had no women as invited speakers. Encouragingly, due to the (slow) change in the landscape and an increased emphasis by the organizers on an improved balance of representation, in 2022, 25% of invited speakers were women (with the proportion of women being the same for contributed talks as well.)

## How do these disadvantages manifest?

The biases and barriers described in the previous section manifest in a range of different measurable ways. It is worth noting that even 1–3% differential effects result in a significant disparity in career progression in the long term, due to cumulative effects^52^, and so addressing these barriers is of utmost importance for fostering an equitable community.

Like their underlying causes, many of these disadvantages can be primary (directly stemming from one of the causes in the previous section) or secondary/tertiary (a resultant effect of historical or institutional inequity). Note that we are not discussing explicit sexism (e.g., inappropriately commenting on a colleague’s physical appearance or personal life), harassment, or abuse. **As a community, we should agree that explicit sexism should be universally condemned, and perpetrators of these actions should be held accountable**. These overt actions are often mistakenly considered the sum-total of sexism in the workplace; here, we choose to elaborate on less clear-cut microaggressions and inequities and the cumulative harm that they cause^53^.

### Recognition

“I am more likely to be dismissed when I contribute. It took a lot longer for my efforts to be recognized than my male counterparts. More credence is given to male coworkers’ ideas who have not proven themselves yet.” (Female software engineer)^34^

“Several times, I was so excited as I made ‘famous people’ in my field ‘wake up’ or pay attention when I took the podium at a conference (because of the novelty of a woman speaking) or spoke in a meeting; only afterwards to hearing through the grapevine that they just commented on my looks. Honestly, it is a very double-edged sword, being different in any way. Immediate attention is yours, but many times—no matter how well you do and how you perform—you can’t sway the inherent prejudice. I imagine that is the same for any field when you are not mainstream.” (Prof. Kristin Persson, in an interview with the Edison Awards, on the support of the field in the context of being a woman)^54^

The under-attribution of women’s contributions to the scientific endeavor is a phenomenon that has been identified and discussed for over 150 years^55^, and is ubiquitous enough to have garnered the name the “Matilda Effect”^56^. **Women often do not receive the same level of recognition for scientific achievements as men, with their contributions more likely to be overlooked, discredited, or attributed to a man**. This under-attribution can manifest in many ways within academia, including the devaluing of women’s contributions to research papers^57^, the association of women authors’ contributions with lower scientific value^24^, the increased likelihood that women will not be credited with authorship for the work they produce (especially if that work has a higher potential to attract significant recognition)^58^, fewer invited papers published by women^59^, and lower rates of success among women in disseminating their work online^60^. The effects of these exclusions are particularly damaging for scientists in the early stages of their careers, for whom recognition often unlocks future opportunities within the broader research community. The following sections detail manifestations of this under-recognition in specific academic contexts.

#### Citation

“I often notice at conferences that as a woman steps onto the podium, several members of the audience open their devices to check her citation record and H-index. This rarely happens to male speakers.” (Male early career researcher)

Citations are one of the primary means by which academics recognize each other’s work^61^. In keeping with the general trend of the under-recognition of women’s contributions to science, papers written by women suffer from lower citation rates with respect to papers written by men across a wide range of fields^62–74^. This discrepancy is more pronounced in fields with a larger gender disparity^74^, or if a man most likely compiled the reference list^73^. In analyzing their own editorial decisions, the Royal Society of Chemistry also found that small gender disparities also exist in the authorship of manuscripts rejected without peer review^74^.

#### Recommendation letters

“When you read confidential letters, you often find that women are nice, have children, and balance work and career. I’m confident that most of the people who write these things truly believe they’re being helpful, but I’m here to say that they are not!” (Judith D. Singer)^44^

Subjective evaluation is a necessary part of recognition – without mechanisms for subjective input, each researcher would be boiled down to citation metrics and funding numbers. However, it was shown that recommendation letters for women researchers are shorter and contain more references to personal traits, fewer to academic achievements, and more “doubt-raisers”, i.e., word or phrase choices that bring into question an applicant’s qualification. These doubt-raisers can be as simple as including irrelevant information or hedging qualifiers (“the candidate *may* make a good colleague”) or they can be as overt as focusing on candidate’s weaknesses, rather than strengths.^75–77^

#### Funding and resources

In a survey led by the International Union of Pure and Applied Physics (IUPAP), female researchers were significantly less likely to feel that they had access to sufficient resources (both monetary and otherwise) to conduct their work than their male counterparts^50^. And it has been shown that the proportion of women grant awardees is lower than the proportion of women applicants^78,79^. Even when overall success rates for men and women are equal, women receive less research funding than men, and are less often listed as principal investigators. Studies show that the language used through the funding allocation process (from the original funding call to instructions and evaluations) is often heavily gendered, with “the quality of researcher” regularly being rated higher for men, even where no gender difference in the “quality of proposal” was found^78^.

#### Awards and prizes

“In the 27 years that this award has been given, I am the first female recipient. While I would not argue with the worthiness or accomplishments of the previous winners, you would be hard-pressed to convince me that there hasn’t been one woman before me who deserved this honor.” (Prof. Sharon C. Glotzer, upon winning the Aneesur Rahman Prize for Computational Physics from the American Physical Society)

Men continue to win a higher proportion of awards for scholarly research than expected based on their representation in the nomination pool (eight times more likely for scholarly awards and three times more likely for young investigator awards)^80^. For example, for awards relevant to our field, where we could collect data on awardees for multiple years: PRACEdays Award (10%), Volker Heine Young Investigator Award finalists (10%). This is the result of the combination of lack of self-promotion and self-nomination of women and lack of seeking out nominations by others. Man-only panels are 50% less likely to choose a female scientist than a panel with one female member, with the gender of the panel chair mattering the most^43,81^.

“Women-only” awards and funding (including schemes for those returning from a career break or having caring responsibilities, which predominantly receive applications from women) were originally structured to address award-based inequities, and many provide scientists with non-traditional backgrounds the opportunity to pursue research and increase their visibility. However, siloing women into restricted categories can have several unintended troubling effects, including gender-restricted awards being perceived as “lesser” versions of non-gender-restricted awards, and can over-inflate the statistics of gender equity in awards^24,80^. For example, the authors of ref. ^80^ noted that gender-based awards caused a 55% inflation in the proportion of American Physical Society honors awarded to women in the 2000s. Quotas can be regarded as tokenism, resulting in a backlash from colleagues and negatively impacting the confidence of women^82^.

#### Student evaluations

“I teach a computational modeling class for undergraduates together with a male colleague. Students regularly address him as ‘Professor’ and address me as ‘Miss’, and once a student told me they had not expected that women could write a code.” (Female assistant professor)

Bias is also present in student evaluations^83^. Female lecturers are more often evaluated based on personality or appearance. As feminine behavioral characteristics are associated with being likeable and approachable, students generally expect more sympathy and social sensitivity from female lecturers^84^, and thus penalize discrepancies from this stereotype, such as ageism, especially for middle-aged women^85^. On the other hand, masculine traits are associated with more competence and better leadership^30^, with male lecturers generally achieving higher student ratings, even in experimental studies that equalize teaching quality^84,86,87^. While evaluation differences seem to have been decreasing over the past decade, it is a worrying trend that in recent years the proportion of abusive student comments have increased, specifically towards women and those from marginalized groups^88^.

### Barriers in working environment

#### Opportunities for networking

Social networks appear to be more beneficial for men, with similar networks resulting in more job and collaboration opportunities for men than for women^89^. Informal social settings are often dominated by men, due to their higher availability (looser caring commitments), choice of setting (venues perceived as male-dominated, e.g. pubs), or women not being invited to avoid misconception of sexual advances. This exclusion from informal research networks^12^ reduces women’s ability to network, feeling of connectedness and inclusion, and ability to gather information through informal channels^34,90^. Furthermore, in male-dominated groups gender-linked interactional behaviors are more likely to emerge. Data has shown that male group-leaders are not only less likely to have female graduate students and postdoctoral researchers in their group, but that the ratio gets worse as the prestige of the group (or the PI) increases^91^. This negatively affects the training and networking opportunities available through the most elite environments.

#### Workload

“There are lots of things expected of you as a woman in academia but there is no equality in terms of what is recognized that contributes to your progression” (Female academic)^11^

“In addition, very often women, especially women in STEM fields, are in high demand for service roles in their home institutions as well as in service to the field. Committees for federal funding agencies, technical societies, and within universities, as well as editorial positions, strive to ensure gender balance. Because there are generally fewer women in STEM fields, this means that a smaller number of women are asked to serve on a large number of these positions. This can be overwhelming for women at every stage of their career.” (Prof. Susan Sinnott, in an interview with *Elsevier*)^92^

While an organizational culture of overwork results in dissatisfaction for both women and men, women are more likely to suffer negative consequences, from poor health to leaving their academic positions^19,93^. Women tend to be asked to do more, more often^94^, and are more likely to agree to (and volunteer to) perform tasks^95^ that are less likely to be taken into account during promotion processes (e.g., mentoring, report writing, and note-taking in meetings)^96^. Women also tend to have higher teaching loads^97^. These responsibilities do not necessarily lead to promotion within academia, further exacerbating gender segregation at higher career stages (or vertical gender segregation).

## Okay... so what can we do?

The first step in addressing the barriers is to acknowledge the existence of bias and that we are all influenced by stereotypes, either consciously or unconsciously. First and foremost, **raising awareness, educating ourselves, participating in training, and learning to challenge others’ and our own perceptions is critical**. Similarly, while creating action plans based on research and evidence is an important tool to facilitate institutional change, holding ourselves accountable for actually implementing action plans is just as crucial^98^.

***Unconscious bias training*** consists of raising awareness of gender biases and working actively to make unconscious biases conscious, so that they can be challenged. It is important that this happens both among men and women and targets both students and established researchers, to facilitate culture change in the long term^99^. Experiential training can change perceptions, raise self-awareness, and teach skills to effectively respond to and avert biased situations. Taking these lessons, we can then more consciously and effectively employ EDI-focused toolkits to improve our practices as educators and researchers^100^.

However, unconscious bias training is not a silver bullet: To be effective, the employed training programs have to be well-designed and evidence-based^101^, and augmented with other positive actions. Implicit assumption tests might be useful tools for augmenting training programs^102^. There are tools available for both identifying our own biased thoughts and gender-based wording in letters, assessments and advertisements, including but not limited to:

- Implicit Bias across Multiple Dimensions
- Gender Bias in Recommendation Letters
- Gender Bias in Job Advertisements

“It will also be important to ensure that biases are fought with and for them at every step of the way, EACH and EVERY time they arise, including subtle biases.” (Prof. Giulia Galli, in an interview with AZoM, on barriers facing women in the field)^103^

### Gender representation metrics

While collecting data in itself does not bring change, having access to good quality statistics is necessary to pinpoint particularly problematic processes or to measure progress in gender representation in research. Accordingly, the collection of information related to gender ratios in various academic contexts (if this can be done anonymously) would contribute to useful benchmarks. For example, it would be useful to quantify and report the proportion of women attending or presenting at conferences, or to publish yearly reports on the gender ratio of authors in scientific journals.

### Changing the narrative

STEM research, especially engineering and computing, is often perceived as unengaged from society and mostly involving solitary work. By showing and advertising how these research areas can contribute to communal goals, we can help dismantle these barriers and make the area more appealing to students of more diverse backgrounds. For example, we can emphasize that a wide variety of expertise is necessary to become a STEM scientist, thereby removing the narrow focus on specific math or computing skills, to fight the perception that women are not naturally a good fit in these STEM research areas^19^.

***Role models*** are critical for encouraging women to join computational materials science and helping to engender a sense of belonging and professional confidence^51,104^. To increase the visibility of women in academic contexts, individuals can strive to draw from a diverse range of examples in teaching, or invite a balanced set of speakers at conferences, seminar series, and panel discussions.

However, until we reach parity, we must remember that a 50–50 representation can put an unrealistic burden on the much smaller number of female researchers. Accommodating flexibility in timing and virtual attendance can help to alleviate some of the burden, in addition to incorporating free childcare facilities at conferences for those lacking alternate support.

**As a community, we should spearhead a multi-national, multi-institution effort to create a database of female and underrepresented computational materials scientists to amplify lesser-known researchers and distribute the efforts put forth in favor of equity**, and individuals should follow those already involved in the effort of highlighting female scientists (e.g., Dr. Jess Wade and the 500 Women Scientists Project or Dr. Adriana Paluszny and the Watson Forum).

***Mentoring*** of young researchers from diverse backgrounds is critical to support those historically excluded from the field. Effective mentoring offers regular contact for an extended time period, with meaningful training offered for mentors^105^. Individuals should promote multi-dimensional mentorship and evaluation in both research and teaching endeavors (for which there are many resources, including ref. ^100^), and institutions should develop and support formalized mentoring programs that extend not only to academic development but also adopt a holistic view of professional growth^106^.

“I would suggest trying to find a good mentor, a person who does not just want you to publish a certain number of papers per year for their celebrity, but that instead really tries to help you grow as a person and teaches you, for example, how to give good presentations, how to write papers, how to deal with these difficult situations, and also how to behave as a scientist.” (Prof. Laura Gagliardi, in an interview with AZoM, on advice for young women in the field)^107^

### Reform of funding and research metrics

We advocate for an innovative reform of the funding landscape to address cumulative bias, such as introducing a “Universal Basic Research grant”, and open discussions on the role and legitimacy of individual awards^43,108^. For all grants and awards, we can actively work on widening the nomination pool, and encouraging a wider set of researchers to nominate. We can also create and publish metrics of nominators and nominees at the end of the selection process (thereby raising awareness, practicing transparency, and tracking our progress in addressing bias)^109^. The recommendations of the San Francisco Declaration on Research Assessment (DORA), which intends to stop the practice of correlating publication and journal impact factor to the merits of an individual scientist’s contributions^110^, are especially salient here. Double-blind peer review can also be a helpful tool to lessen the effect of biases^111^.

***Improving the working environment*** consists of ensuring that job responsibilities can be adjusted (part time, flexible working, leave policy) without carrying a stigma^93^, and that adjustments are made available irrespective of the staff member’s gender. Inclusiveness at the workplace must also extend to informal social interactions, to foster a shared identity and improve career outcomes for women^90^.

For family responsibilities^12^, adjustments consist of providing adequate on-site childcare facilities, promoting shared parental-leave policies, and creating institutional policies for modified duties (with a reduction of non-promotable workload)^112,113^. **Sufficient parental leave and professional responsibility adjustments are critical pillars of institutional support**, not only for mothers but for all parents, as well as the cultivation of the norm that *both* parents make use of these policies^46–48^. We point interested readers to the excellent book “Do Babies Matter?: Gender and Family in the Ivory Tower”, for a focused discussion on the topic^46^.

“I can just advise young women to think for themselves, and be confident in what they do not to give up their dreams too easily. And don’t let anybody tell them that they have to choose between a career and a family.” (Prof. Dr. Silvana Botti, in an interview with Friedrich Schiller University Jena, on life balance)^114^

### Citation metrics

The first step toward citation equity consists of a transparent disclosure and analysis of citation behavior at the individual author and collective journal levels. To this end, we advocate for the inclusion of a citation diversity statement (CDS) at the conclusion of papers, in a similar manner to the inclusion of funding acknowledgments or statements of conflicts of interest. The CDS is a simple acknowledgment of the importance of citation diversity and a self-reporting of the gender-breakdown of a paper’s citations^115^. It effectively places each paper in the gendered context of the knowledge base on which it relies, and serves as a tool through which authors and readers can examine their own citation practices. A number of journals have embraced the CDS, including the *Journal of Cognitive Neuroscience*^70^ and all journals published by the Biomedical Engineering Society^116^. Editors at the *Journal of Cognitive Neuroscience* reported a clear reduction of gender imbalances in citations in the year following the journal’s embrace of the CDS^117^. For readers interested in calculating and reporting their own citation statistics, several publicly available tools exist that are both journal-specific^70^ and journal-general^118^.

## Final remarks

Gender equity is a nuanced and complex topic, with enough aspects to fill several graduate theses. We don’t set out to be exhaustive on this topic, but to provide summaries and resources for those seeking them within the field of computational materials research. For the interested reader, we suggest similar perspectives from the field of Neuroscience^9,119^, or the comprehensive reports of the American Association of University Women^19^ and the Workshop on Gender Equity in Materials Science and Engineering^12^.

We also note that this perspective is but one (incomplete) lens through which to view broader issues of bias and discrimination within STEM. Left out of this conversation are scientists with intersecting marginalized identities. Trans and non-binary scientists, for example, face unique challenges not fully covered by our discussion. We look forward to future work that will foreground these communities and push academia toward an ever-more equitable landscape.

## Citation diversity statement

In writing this paper, we sought to proactively consider choosing references that reflect the diversity of the field in thought, form of contribution, gender, and other factors. We use databases that store the probability of a name being carried by people of different genders to mitigate our own citation bias at the intersection of name and identity. Based on the databases used, the set of names assigned the “woman” label will contain a predominance of women and the set of names assigned the “man” label will contain a predominance of men, but both sets may also contain other genders. By this measure (supplemented by manual research of some individual authors and excluding self-citations to the first and last authors of our current paper, and papers whose authors’ first names could not be determined), our references contain 46.1% woman(first author)/woman(last author), 15.7% man/woman, 24.5% woman/man, and 13.7% man/man categorization. This method is limited in that names, pronouns, and social media profiles used to construct the databases may not, in every case, be indicative of gender identity. Furthermore, probabilistic studies of names cannot be used to detect citation costs that are specific to intersex, non-binary, or transgender people who are out to a large number of their colleagues. We look forward to future work that could help us to better understand how to support equitable practices in science.
